# Evidence for a transcellular route for vitellogenin transport in the telotrophic ovary of *Podisus nigrispinus* (Hemiptera: Pentatomidae)

**DOI:** 10.1038/s41598-019-52789-z

**Published:** 2019-11-11

**Authors:** Mírian Quintão Assis, Virgínia Teles Dohanik, Leandro Licursi de Oliveira, José Cola Zanuncio, José Eduardo Serrão

**Affiliations:** 1 Department of General Biology, Universidade Federal de Viçosa, 36570-000 Viçosa, Brazil; 2 Departament of Entomology, Universidade Federal de Viçosa, 36570-000 Viçosa, Brazil

**Keywords:** Electron microscopy, Entomology

## Abstract

Vitellogenin is the main yolk precursor protein in insect oocytes. It is synthesized in the fat body and released into the hemolymph. To reach the oocyte surface, vitellogenin must cross a single layer of follicular epithelium cells. The transport of vitellogenin across the follicular epithelium has been suggested to occur through the enlarged intercellular spaces (patency) by a paracellular route or by endocytosis by follicular cells and release onto oocyte surface in a transcelluar route. In this study, we investigated whether vitellogenin transport in the meroistic telotrophic ovary of *Podisus nigrispinus* (Hemiptera) occurs via a paracellular or transcellular route. Light and transmission electron microscopies showed that short cell–cell contacts with well-developed occluding septate junctions were present in follicular cells with patency. Immunofluorescence microscopy revealed the presence of vitellogenin receptors in the plasma membrane and of vitellogenin in the cytoplasm of follicular cells. Data suggest that cell–cell contacts serve as a barrier to large vitellogenin molecules and that this protein is transported via a transcellular route of receptor-mediated endocytosis.

## Introduction

The female insect reproductive tract is formed by a pair of ovaries connected to the common oviduct by a pair of lateral oviducts. Each ovary has many functional units, the ovarioles, in which oocytes grow surrounded by a follicular epithelium^1^.

In Heteroptera (Hemiptera), which includes bugs, ovaries are of the meroistic telotrophic type^2–4^ and each ovariole is divided into terminal filament, tropharium, vitellarium, and pedicel^3,5^. The distal portion of the ovariole is composed of the terminal filament, formed by connective tissue^6^; a tropharium with oogonia (which differentiate into oocytes) and nurse cells, which produce cytoplasm compounds that are transferred to oocytes during development^5–7^.

Oocyte maturation and vitellogenesis occur in the vitellarium^2,8^. Vitellogenesis is crucial for insect reproduction and comprises the synthesis of vitellogenin and yolk accumulation in the ooplasm^9^. Synthesis of the glycolipophosphoprotein vitellogenin occurs in the fat body under juvenile hormone control^2,10^. Vitellogenin is released into the hemolymph, taken up by growing oocytes, and stored as vitellin, the major yolk protein^11,12^.

Vitellogenin uptake into oocytes occurs via endocytosis, mediated by membrane vitellogenin receptors (VgRs) belonging to the low-density lipoprotein receptor (LDLR) superfamily^9,11,13–15^. LDLRs shares some structural features such as ligand-binding domain (LBD), epidermal growth factor precursor (EGFP) homology domain, O-linked sugar domain, a single transmembrane domain and a cytoplasmic domain^14–16^. Insects have two LBDs with different numbers of class A cysteine-rich ligand binding repeats (LBRs)^16–20^. These VgRs have been reported to combine with some different ligands in vertebrates^21^, and their mRNA are also detected in the hypopharingeal glands^22^ and fat body^16,23^ in insects, suggesting that VgR may recognize other ligands, but in insect ovary vitellogenin is the unique ligand of VgR endocytosed by oocytes^11,13–15,24^. However, to reach the oocyte surface, vitellogenin must be transported through the follicular epithelium^25^, which surrounds the oocyte^2,6^ and regulates the flow of vitellogenin to the oocyte^5,7^.

A model proposed over fifty years ago to explain the vitellogenin transport through the follicular epithelium remains almost intact. Extracellular canals formed by enlargement of the intercellular spaces between the follicular cells (patency) facilitate diffusion of soluble proteins from the hemolymph until the oocyte surface via a paracellular route. Transmission electron microscopy and immunohistochemical studies in *Hyalophora cecropia* (Lepidoptera)^26–28^ and *Bemisia tabaci* (Hemiptera)^29^, dynamics of the vital dyes trypan blue in *Aedes aegypti* (Diptera)^30^ and Evan’s blue in *Rhodnius prolixus* (Hemiptera)^31^ suggest the occurrence of paracellular transport of proteins trough the ovarian follicular epithelium. Conversely, in social Hymenoptera with meroistic polytrophic ovaries, ultrastructural studies indicate patency in the follicular epithelium^25^, but immunocytochemistry of vitellogenin and VgR show that both are localized in the plasma membrane and cytoplasm of follicular cells, suggesting a transcellular route in which vitellogenin initially diffuses through intercellular spaces to the follicular cells surface, bind to VgR, following clathrin-mediated endocytosis and transport to another plasma membrane domain (transcytosis) closely associated with oocyte surface^32^. This recent finding stimulated us to verify whether in other insects with meroistic telotrophic ovaries, patency is already an evidence of paracellular route for the vitellogenin transport to the oocyte surface for further endocytosis.

*Podisus nigrispinus* (Hemiptera: Pentatomidae) is an important generalist predator used in biological control of agricultural insect pests^33^. The development^34^, histology, cytology^35–37^, predator–prey interaction^38^, and biochemical processes^39^ of this predator have been studied. However, to the best of our knowledge, data on the morphology and reproductive physiology of *P*. *nigrispinus* females are scarce^4,40^ and the route for vitellogenin transport from the hemolymph to the oocyte surface has yet to be determined.

The objective of this study was to investigate whether vitellogenin follows a paracellular or transcellular route through the follicular epithelium in *P*. *nigrispinus* and thus contribute to the comprehension of this insect’s reproduction cycle.

## Results

**Figure 1:**
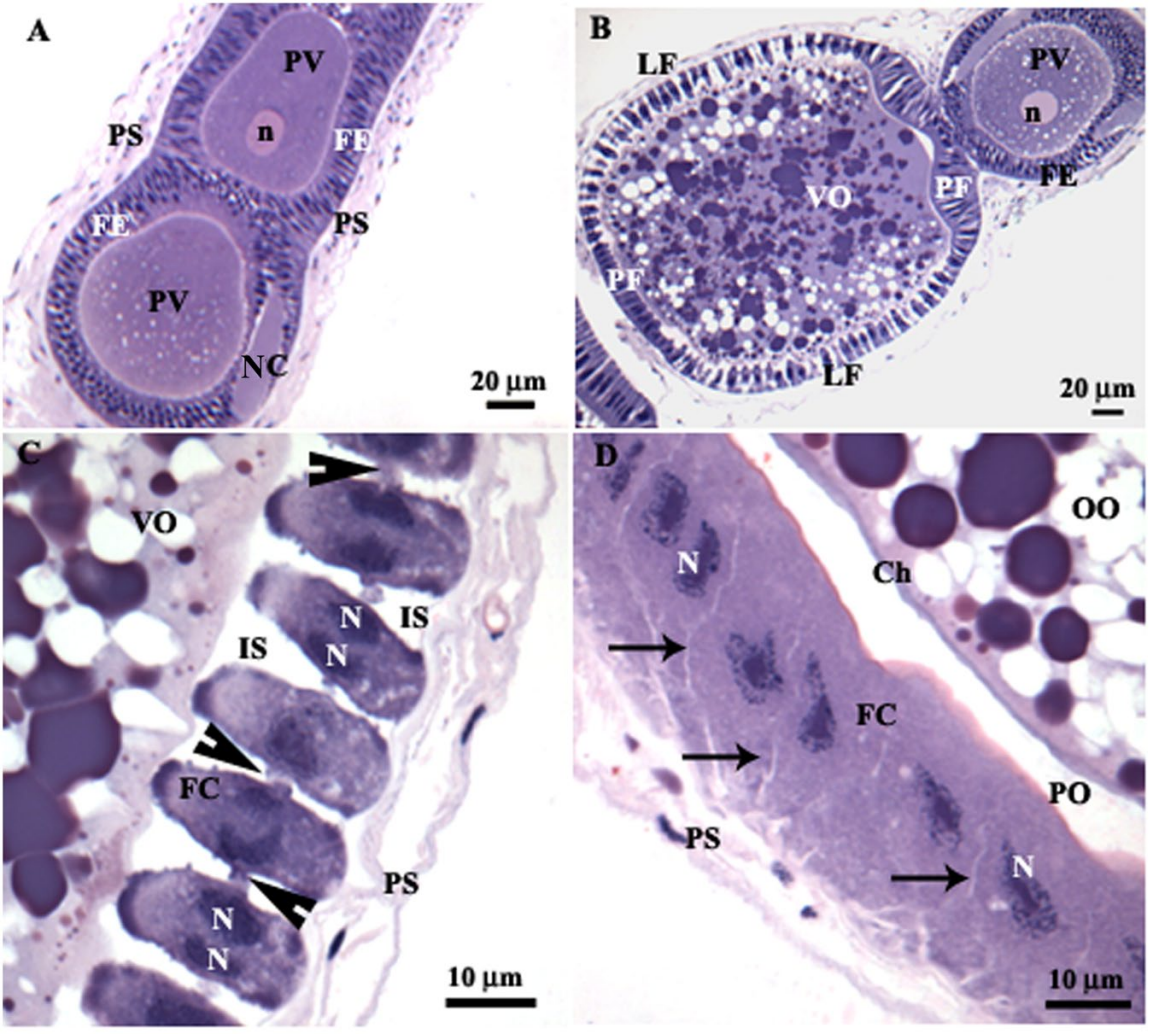
Light micrographs of the ovariole of *Podisus nigrispinus*. (**A**) Previtellogenic oocytes (PV) with homogeneous ooplasm and nucleus (n) lined by follicular epithelium (FE) and peritoneal sheath (PS). (**B**) Vitellogenic oocyte (VO) with ooplasm showing many yolk granules. Note polar follicular epithelium (PF) with columnar and juxtaposed cells and lateral follicular epithelium (LF) with enlarged intercellular spaces. (**C**) Lateral follicular epithelium of vitellogenic oocyte (VO) showing binucleated (N) cells with enlarged intercellular spaces (IS) and small points of cell-cell contact (arrowheads). (**D**) Late vitellogenic oocyte (OO) showing chorion (Ch), perioocytic space (PO) and follicular cells (FC) with narrowed intercellular spaces (arrows). NC – nutritive cord.

*Podisus nigrispinus* oocytes in the vitellarium were enveloped by a single layer of binucleate follicular cells (Fig. 1A). Previtellogenic oocytes were characterized by their small size and homogeneous cytoplasm, lined by columnar follicular cells with narrow intercellular spaces (Fig. 1A).

During vitellogenesis, oocytes increased in size and had many yolk granules and lipid droplets in the cytoplasm (Fig. 1B). Follicular cells at the anterior and posterior poles of the ovarian follicles were columnar and juxtaposed (Fig. 1B), whereas those in lateral regions were cubic and separated by enlarged intercellular spaces (Fig. 1B,C) but always showed small points of contact (Fig. 1C).

In advanced vitellogenesis, the perioocytic space (between oocyte surface and follicular cells) increased in size, eggshell (chorion) synthesis occurred, and intercellular spaces of follicular cells narrowed (Fig. 1D).

**Figure 2:**
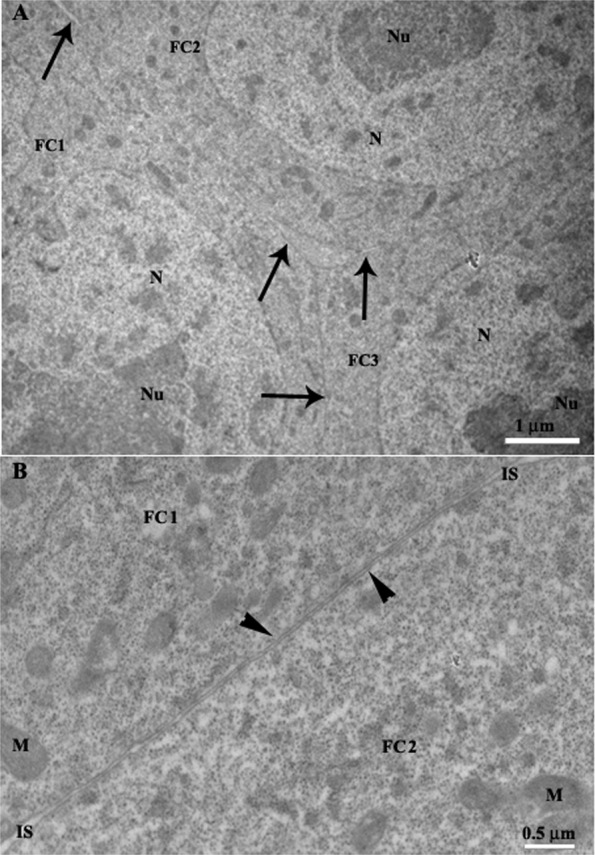
Transmission electron micrographs of the follicular epithelium of the previtellogenic follicles in the ovarioles of *Podisus nigrispinus*. (**A**) Three adjacent follicular cells (FC1, FC2 and FC3) showing narrowed intercellular spaces (arrows). N – nucleus, Nu – nucleolus. (**B**) Detail of two adjacent follicular cells (FC1 and FC2) showing plasma membrane (arrowheads) and narrowed intercellular spaces (IS). M – mitochondria.

**Figure 3:**
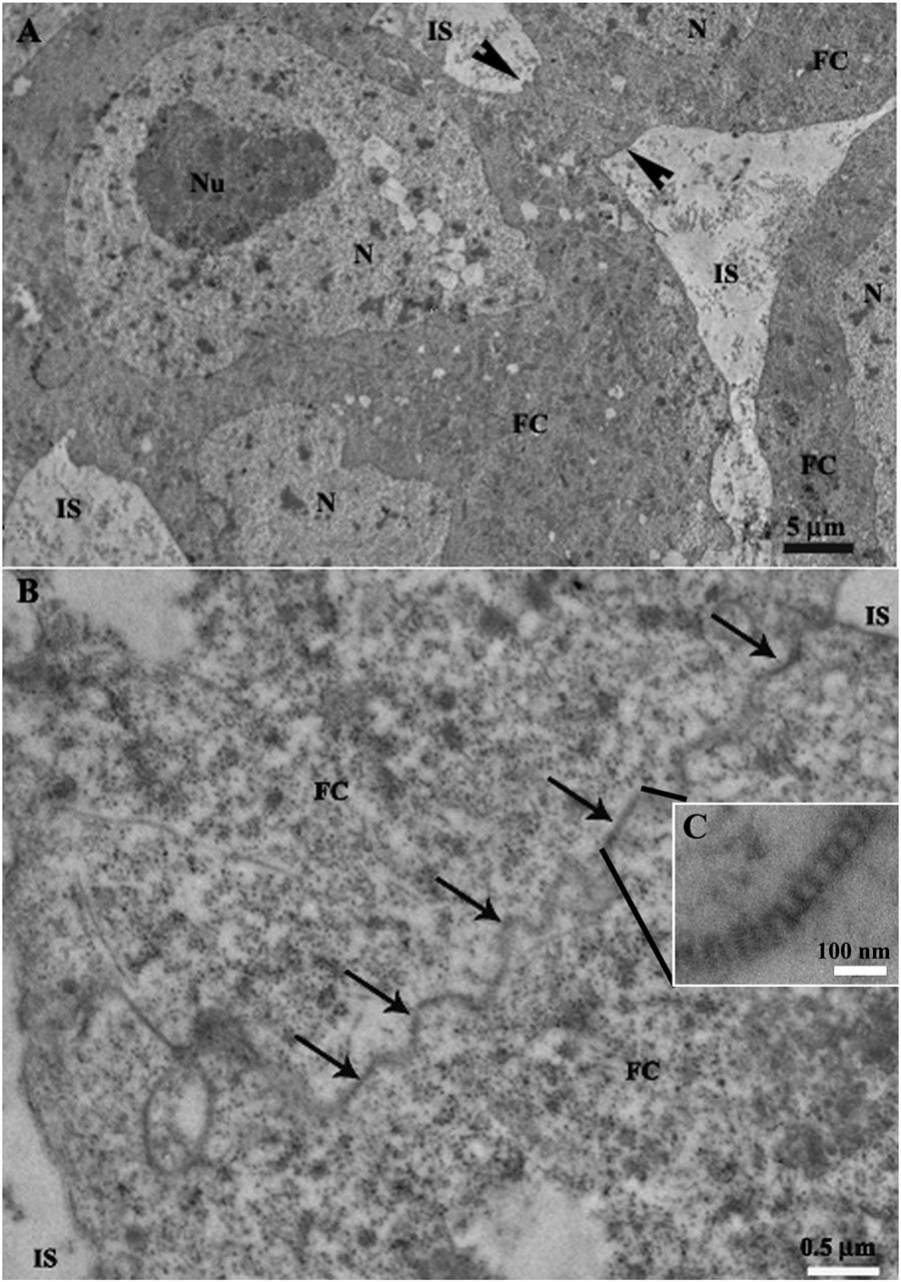
Transmission electron micrographs of the vitellogenic follicles in the ovarioles of *Podisus nigrispinus*. (**A**) Adjancent follicular cells (FC) showing enlarged intercellular space (IS) and a small point of cell-cell contract (arrowheads). (**B**) Region of follicular cell-cell contact showing narrowed intercellular space with many occluding junctions (arrows). (**C**) Detail of the occluding junction with parallel rows characterizing septate junction. N – nucleus, Nu – nucleolus.

The ultrastructure of follicular cells during vitellogenesis in Hemiptera was previously described^2^. Therefore, we used transmission electron microscopy to evaluate intercellular contacts between follicular cells in previtellogenic and vitellogenic follicles. In the former, follicular cells showed narrow intercellular spaces along almost all their extension (Fig. 2A) as well as some septate junctions (Fig. 2B). In the vitellogenic follicle, there were enlarged intercellular spaces, which were interrupted by short contacts between adjacent follicular cells (Fig. 3A). At these cell–cell contact points, the intercellular space was narrow and plasma membranes showed septate junctions along almost the entire length (Fig. 3B,C).

**Figure 4:**
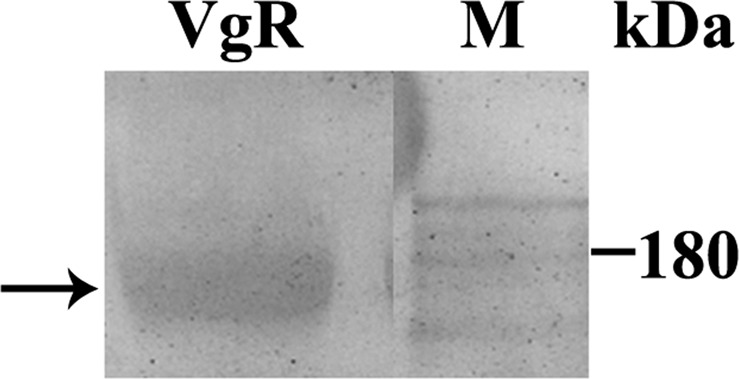
Western blotting of membrane proteins extracted from ovaries of *Podisus nigrispinus* using anti-VgR antibody showing detection of VgR (arrow, 180 kDa). M – Molecular marker.

**Figure 5:**
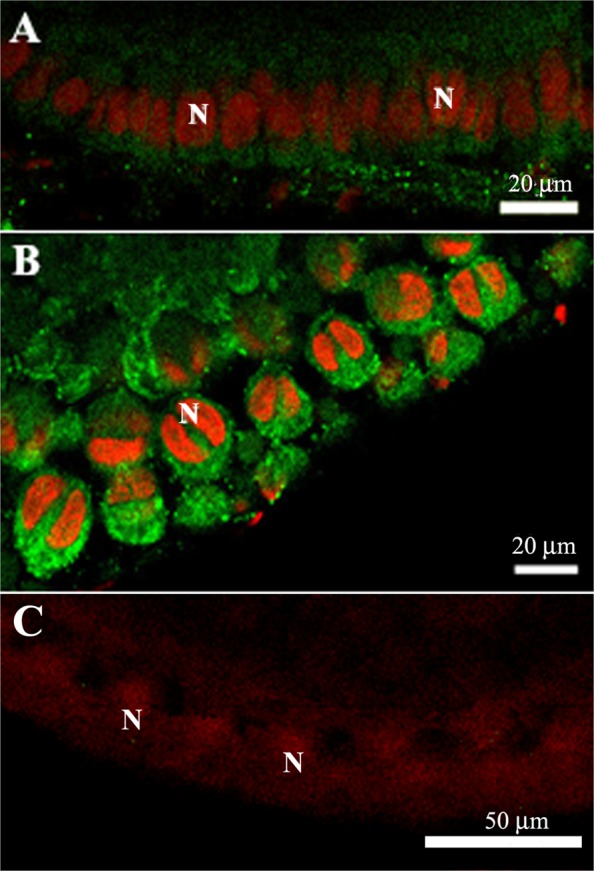
Immunofluorescence staining of follicles of ovarioles of *Podisus nigrispinus*. (**A**) Occurrence of vitellogenin receptor (green) in follicular cells of the previtellogenic follicle. (**B**) Vitellogenin receptor (green) in follicular cells of the vitellogenic follicle. (**C**) Negative control by omission of incubation with vitellogenin receptor antibody. Nuclei (N) of follicular cells in red.

**Figure 6:**
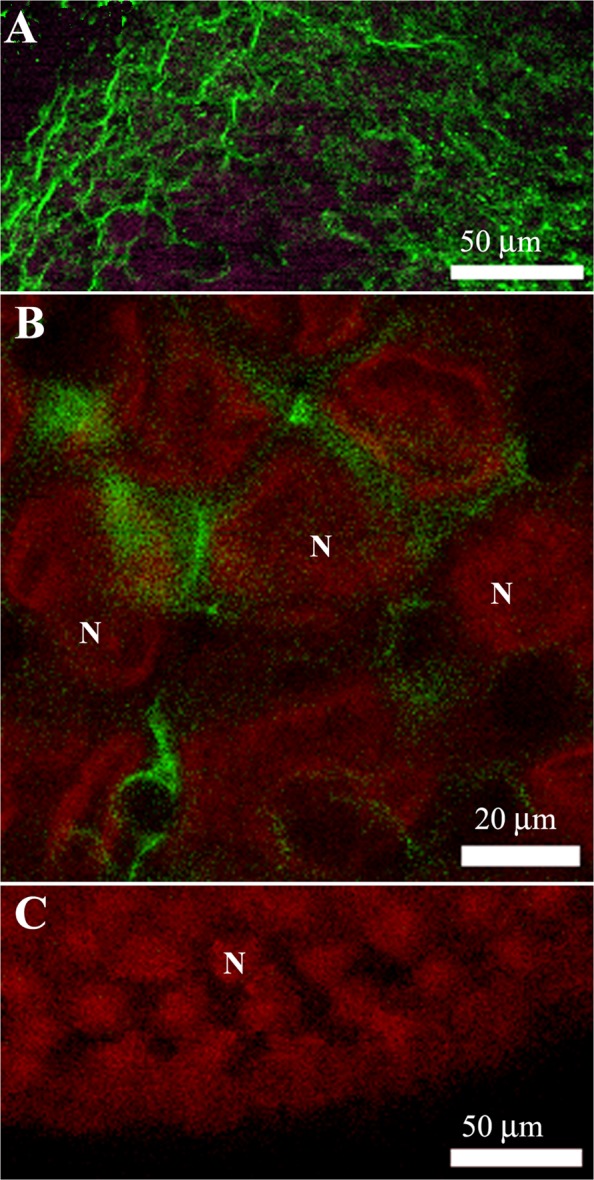
Immunofluorescence staining of vitellogenic follicles of ovarioles of *Podisus nigrispinus*. (**A**) Occurrence of vitellogenin (green) in follicular cells of the vitellogenic follicle. (**B**) Detail of vitellogenin (green) in the follicular cell. (**C**) Negative control by omission of incubation with vitellogenin antibody.Nuclei (N) of follicular cells in red.

To investigate whether vitellogenin is transported to the perioocytic space via a paracellular or transcellular route, we analyzed previtellogenic and vitellogenic follicles using immunofluorescence microscopy for detection of VgR. Western blotting showed that the antibody used recognized VgR, confirming the presence of this protein in the plasma membrane of *P*. *nigrispinus* oocytic follicles (Fig. 4). Immunofluorescence microscopy revealed the presence of VgR (Fig. 5) on the surface of follicular cells in both previtellogenic (Fig. 5A) and vitellogenic follicles, with a high fluorescent signal in the latter (Fig. 5B). Since we found high amount of VgR in the vitellogenic follicle, these were evaluated for the uptake of the vitellogenin, showing presence of this protein into the follicular cells (Fig. 6).

## Discussion

Our findings show that the follicular epithelium in vitellogenic follicles of *P*. *nigrispinus* is differentiated into columnar and juxtaposed cells at the anterior and posterior poles, whereas follicular cells in lateral regions are cubic with dilated intercellular spaces. A similar morphology was observed in *R*. *prolixus* (Hemiptera) at the onset of vitellogenesis, when cells of the follicular epithelium at the anterior and posterior poles are morphologically distinct from cells in the lateral portion^41^. The intercellular spaces between follicular cells of the epithelium lateral to the oocyte enlarge progressively with oocyte growth, whereas polar follicular cells continue to possess narrow intercellular spaces and might act as a barrier for the passage of yolk proteins^42,43^, but the physiological significance of this difference remains unknown.

Enlargement of intercellular spaces in the follicular epithelium (patency) during vitellogenesis, as observed in the present study in *P*. *nigrispinus*, has been reported in *H*. *cecropia*^26,27^, *R*. *prolixus*^28,41^ and *B*. *tabaci*^29^. Those studies show accumulation of hemolymph proteins in the intercellular spaces of follicular cells, which has been interpreted as an evidence that these proteins, including vitellogenin, are transported to the oocyte surface via a paracellular route. However, in bees, ants, and wasps, although patency occurs, vitellogenin is transported to the oocyte surface by receptor-mediated endocytosis, that is, via a transcellular route^25,32^.

Light and transmission electron microscopies showed that patency occurs in the follicular epithelium of *P*. *nigrispinus* vitellogenic follicles but also revealed that follicular cells display septate junctions in small cell–cell contact regions. Septate junctions play an important role in maintaining the mechanical and physiological integrity of the epithelium^41,43,44^. In addition, septate junctions are important permeability barriers that control the transit of molecules through invertebrate epithelia^45–48^. Some chemical species such as lanthanum ions (La^3+^), which have high charge density and occur in a hydrated state, can cross septate junctions^43,49,50^; however, the passage of vitellogenin, which is a large protein with a molecular mass of 200–700 kDa^14,51^, seems unlikely, as septate junctions in different epithelia of insects are effective barriers to some ions and molecules larger than 7 kDa^49,52–54^.

Together, ultrastructural and immunofluorescence data show that intercellular spaces are blocked by septate junctions and that the plasma membrane of follicular cells contains VgR and vitellogenin, suggesting a transcellular route of vitellogenin transport to the perioocytic space, as has been demonstrated in Hymenoptera^32^. However, the question remains as to why patency occurs in follicular cells of insects during vitellogenesis. A possible explanation would be that the almost complete separation of adjacent follicular cells allows the exposure of a larger area of the plasma membrane to the hemolymph and thus exposes many VgR molecules for vitellogenin uptake, resulting in a high transport rate of reserve proteins to the oocyte.

The present study provides insight into the transcellular route of vitellogenin transport through the follicular epithelium in meroistic telotrophic ovaries, which seems to be a conserved route in insects, as it also occurs in insects with meroistic polytrophic ovaries. Further investigation is needed to elucidate the routes of vitellogenin transport in insects with panoistic ovaries.

## Methods

### Insects

Mated *P*. *nigrispinus* females were obtained from the Biological Control Laboratory of the Federal University of Viçosa (UFV), Viçosa, Minas Gerais, Brazil.

### Light microscopy

Insects were cryoanesthetized at −4 °C for 90 s, and their ovaries were dissected in 125 mM NaCl. Five pairs of ovaries were transferred to 4% paraformaldehyde in 0.1 M phosphate buffered saline (PBS), pH 7.2. Then, samples were washed in the same buffer, dehydrated in a graded ethanol series (70, 80, 90, and 95%), and embedded in historesin (Leica). Sections of 2 μm, obtained using a rotary microtome with a glass knife, were stained with hematoxylin and eosin and analyzed under a light microscope.

### Transmission electron microscopy

Five females were cryoanesthetized at −4 °C for 90 s and dissected. The ovaries were transferred to 2.5% glutaraldehyde in 0.1 M sodium cacodylate buffer, pH 7.2, for 12 h. After being washed in the same buffer, samples were post-fixed in 1% osmium tetroxide for 2 h, dehydrated in a graded ethanol series (70, 80, 90, 95, and 98%), and embedded in LR White resin. Ultrathin sections, obtained using an ultramicrotome equipped with a glass knife, were stained with 2% aqueous uranyl acetate and lead citrate and analyzed using a transmission electron microscope (Zeiss LIBRA 120).

### Western blot analysis

*Podisus nigrispinus* females were dissected, and the ovaries were homogenized in 50 mM Tris-HCl buffer, pH 7.5, containing 10% protease inhibitor cocktail (P2714-1BTL, Sigma–Aldrich) using a cordless motor. Procedures were performed in triplicate. Then, samples were centrifuged at 10,000 × *g* for 15 min, and the supernatant containing soluble proteins was collected. The pellet was resuspended in Tris-HCl buffer, pH 7.5, 1% Triton X-100, and 10% protease inhibitor, homogenized, placed in an ultrasonic bath for extraction of membrane proteins, and centrifuged at 10,000 × *g* for 15 min. Supernatants containing soluble membrane proteins were submitted to SDS-PAGE^55^. The gel was incubated in transfer buffer [0.58% (w/v) Tris-HCl, 0.28% (v/v) glycine, 20% (v/v) methanol, and ultrapure water] and transferred to a nitrocellulose membrane by electroblotting at 190 A and 4 °C for 3 h (Mini Trans-Blot Cell^®^, Bio-Rad). Subsequently, the nitrocellulose membrane was incubated in Tris-buffered saline (TBS) (25 mM Tris and 150 mM NaCl, pH 7.5) with 1% Tween 20 (TBST) and 5% skimmed milk powder for 16 h. The nitrocellulose membrane was rapidly washed with TBST, washed twice with TBST for 15 min, and incubated for 2 h with primary antibodies against vitellogenin receptor (anti-VgR)^32^ (1:500). After washing with TBST, the nitrocellulose membrane was incubated with peroxidase-conjugated anti-mouse IgG secondary antibody (1:100) for 2 h. Then, samples were revealed with a diaminobenzidine solution [0.1% (w/v) DAB, 50 mM Tris-HCl, and H_2_O_2_].

### Immunofluorescence microscopy

After dissection, the ovarioles had the peritoneal sheath removed with tweezers and were immersed in Zamboni fixative [4% (w/v) paraformaldehyde and 0.4% (w/v) picric acid in 0.1 M PBS, pH 7.2] for 2 h. Samples were transferred to PBS with 0.05% Triton X-100 (PBST) for 2 h and incubated in anti-VgR primary antibody^32^ (1:100 dilution in PBST) for 16 h. Another set of ovarioles were incubated in anti-vitellogenin primary antibody^10^ (1:100 dilution in PBST) for 16 h. Then, samples were incubated in a 1:400 dilution of FITC-conjugated anti-mouse IgG in PBST for 2 h in the dark. Samples were transferred to 50 μL of TO-PRO-3 iodide (Thermo Fisher Scientific) in PBS (1:1000), incubated for 30 min in the dark, mounted on slides with 50% sucrose., and analyzed on a Zeiss 510 Meta confocal fluorescence microscope.

Negative controls were subjected to the same procedures, except that the primary antibodies were replaced with normal mouse (for VgR) or rabbit (for vitellogenin) serum.

### Ethical statement

This article does not contain any studies with animals and humans participants performed by any of the authors.

## Data Availability

All data and protocols used in this study are available at Universidade Federal de Viçosa.
